# Yellow Fever Board of Experts

**Published:** 1879-01

**Authors:** 


### Editorial.

### YELLOW FEVER BOARD OF EXPERTS.

Since our last issue, in which we noticed the yellow fever commission's report, the yellow fever Congressional committees of the Senate and House have appointed a board of experts to investigate the cause and spread of cholera and yellow fever. The board organized at Memphis and adopted a plan of investigation similar to that pursued by the commission last October. Dr. Woodworth, Surgeon General of Marine Hospital service, the creator of the commission, is chairman of the board of experts. Two members of the commission, Drs. Bemis and Cochran, are on the board. We do not *know* the views of the other six members, or that they have any settled and expressed opinion as to the origin and spread of yellow fever. We would fain hope they have, at least, no prejudices on the subject which will control them in their action. With Dr. Woodworth at the head, however, and two of the commission as leading members of the Board, we fear that not only the same plan of work will be adopted, but that a similar report will be made.

We must be allowed to enter our plea for unprejudiced, disinterested enquiry into *all* the facts, which will go to prove the true origin of yellow fever in this country.

Let us suggest that facts should be given going to show that the fever has local cause, and that it will not "*spread*," (as the commission, president and all are pleased to term it) without the local cause which produces it. These should certainly be considered as well as those which *seem to favor* importation. Would it not be well to state the facts that excessive rains during summer in the Mississippi valley caused overflow of streams, and that the water in marshes, pools, ponds, lakes and bayous gradually subsided under the most intense heat of summer known to that country? The condition in this respect of Winona, Grenada and all other infected places, would probably show local cause for the fever that prevailed. Memphis particularly requires investigation of this kind. If money is to be spent for the protection of the people against the ravages of yellow fever, this is a rich field for the exercise of government philanthropy. Can the bayou or lake contiguous to this devoted city be drained so that it will not retain the washings of the fertile Mississippi valley which have been suffered to ebb gradually under a burning summer's sun heretofore?

Another important fact will strike every one with force, who has not quarantine on the brain: The refugees from Charleston, Augusta, Savannah, Brunswick, New Orleans and Memphis, have been known to take the fever, after leaving home, at various non-malarious, healthy parts of the country, without communicatiug the disease to others in a single well authenticated instance. In 1834, 1858, 1876 and 1878 persons fleeing their infected homes had the disease in Atlanta, bringing with them packages and trunks of clothing, etc. With these so-called causes of the disease many of our citizens have been brought into constant contact, but in no instance has a case occurred from fomites in clothing or personal communication. Not only has the disease failed to "spread" by transportation, but imported cases of the fever from abroad never communicate the disease, as proven by the known fact that the disease never "spreads" in neighborhoods free from local malarial cause. The Board of experts, and Congressional Committees can be supplied on application with an overwhelming amount of incontestable proof on these points.

If the United States' government seeks to obtain all the facts necessary to determine the true origin of yellow fever, in order to aid in protecting her people against it, why are not some such men put on the Board of experts, as Kinloch, of Charleston, LeHardy, of Savannah, or some of the physicians of New Orleans who have evidences of local origin? At any rate, why are not the facts they publish, going to prove local origin, mentioned in the commission's report?

Now, in conclusion, we have this to say: If, under the leadership of the determined and managing apostle of quarantine, the work of protection is to be carried on by sea, the *form* of investigation now going on, and the expense incurred thereby, might well be dispensed with.

